# A CRISPR/Cas9-based kinome screen identifies ErbB signaling as a new regulator of human naïve pluripotency and totipotency

**DOI:** 10.1093/lifemedi/lnad037

**Published:** 2023-10-20

**Authors:** Jiayu Li, Xiwen Lin, Liangfu Xie, Jingru Zhao, Chunsheng Han, Hongkui Deng, Jun Xu

**Affiliations:** Department of Cell Biology, School of Basic Medical Sciences, Peking University Stem Cell Research Center, Peking University Health Science Center, Peking University, Beijing 100191, China; State Key Laboratory of Stem Cell and Reproductive Biology, Institute of Zoology, Chinese Academy of Sciences, Beijing 100101, China; Institute for Stem Cell and Regeneration, Chinese Academy of Sciences, Beijing 100101, China; Beijing Institute for Stem Cell and Regenerative Medicine, Beijing 100101, China; MOE Engineering Research Center of Regenerative Medicine, School of Basic Medical Sciences, State Key Laboratory of Natural and Biomimetic Drugs, Peking University Health Science Center and the MOE Key Laboratory of Cell Proliferation and Differentiation, College of Life Sciences, Peking-Tsinghua Center for Life Sciences, Peking University, Beijing 100191, China; MOE Engineering Research Center of Regenerative Medicine, School of Basic Medical Sciences, State Key Laboratory of Natural and Biomimetic Drugs, Peking University Health Science Center and the MOE Key Laboratory of Cell Proliferation and Differentiation, College of Life Sciences, Peking-Tsinghua Center for Life Sciences, Peking University, Beijing 100191, China; State Key Laboratory of Stem Cell and Reproductive Biology, Institute of Zoology, Chinese Academy of Sciences, Beijing 100101, China; Institute for Stem Cell and Regeneration, Chinese Academy of Sciences, Beijing 100101, China; Beijing Institute for Stem Cell and Regenerative Medicine, Beijing 100101, China; Savaid Medical School, University of Chinese Academy of Sciences, Beijing 101408, China; MOE Engineering Research Center of Regenerative Medicine, School of Basic Medical Sciences, State Key Laboratory of Natural and Biomimetic Drugs, Peking University Health Science Center and the MOE Key Laboratory of Cell Proliferation and Differentiation, College of Life Sciences, Peking-Tsinghua Center for Life Sciences, Peking University, Beijing 100191, China; Department of Cell Biology, School of Basic Medical Sciences, Peking University Stem Cell Research Center, Peking University Health Science Center, Peking University, Beijing 100191, China

**Keywords:** totipotency, naïve pluripotency, hEPSCs, CRISPR screens, kinome

## Abstract

Regulation of totipotency and naïve pluripotency is crucial for early human embryo development. However, the mechanisms of naïve pluripotency and totipotency regulation in humans, especially the signaling pathways involved in these processes, remain largely unknown. Here, using the conversion of human extended pluripotent stem cells (hEPSCs) to naïve pluripotent stem cells as a model, we performed a CRISPR/Cas9-based kinome knockout screen to analyze the effect of disrupting 763 kinases in regulating human naïve pluripotency. Further validation using small molecules revealed that the inhibition of ErbB family kinases promoted the transition of hEPSCs to human naïve pluripotent stem cells. More importantly, chemical inhibition of the ErbB family also promoted induction of totipotent signatures in human pluripotent cells under different culture conditions. Our findings provide new mechanistic insights into the regulation of naïve pluripotency and totipotency in humans.

## Introduction

The dynamic regulation of cell potency during early preimplantation development is crucial for individual development. In humans, zygotes and early blastomeres, such as blastomeres from 8-cell (8C) embryos are considered to be totipotent, possessing the ability to generate the entire individual [1, 2]. Their developmental potential gradually becomes restricted after differentiating into epiblast, trophectoderm, and primitive endoderm at the blastocyst stage. Intriguingly, unlike their mouse counterparts, human epiblast cells from preimplantation blastocysts still possess both embryonic and extraembryonic developmental potentials [3], suggesting great interspecific differences in the regulation of cell potency during preimplantation development between mice and humans. Although recent studies have revealed several important totipotent transcription factors (TFs) in humans [4–6], the mechanisms of totipotency regulation in humans, especially the signaling pathways that are involved in these processes, remain largely unknown. In addition, despite that the regulatory network of mouse naïve pluripotency has been extensively studied and well understood [7], current understanding of human naïve pluripotency regulation is still limited [8].

Human naïve pluripotent stem cells have emerged as a valuable model for investigating human naïve pluripotency and totipotency *in vitro*. These cells, unlike formative and primed pluripotent stem cells that are derived from peri- and post-implantation stages, respectively [9–11], exhibit functional and molecular features that closely resemble those of preimplantation human epiblast cells [12–14]. Furthermore, human naïve pluripotent stem cells possess the ability to generate extraembryonic lineages [3, 15]. Notably, our previous studies showed the establishment of human extended pluripotent stem cells (hEPSCs) that also exhibit extraembryonic developmental potentials [16, 17]. However, hEPSCs differ from human naïve pluripotent stem cells in terms of their molecular features, as they occupy an intermediate state between the naïve and primed states [17]. In addition, the ability of hEPSCs to generate trophectodermal lineages is not equivalent to that of human naïve pluripotent cells, as human blastoids generated from human EPSCs only recapitulate several molecular aspects of human trophectoderm [18]. Another major difference is that totipotent-like cells are presented in the cell culture of human naïve pluripotent cells [19, 20], which was not observed in hEPSC cultures [20]. Clarifying the mechanisms underlying these differences would not only reveal key molecular targets that regulate the above differences between hEPSCs and human naïve pluripotent cells but also unravel novel regulators of human naïve pluripotency and totipotency, which is important for understanding the mechanistic regulation of cell potency.

Among different factors that affect cell potency, components in signaling pathways play important roles as their manipulation has been proven to be powerful in regulating cell potency *in vitro* [21]. In signaling pathway regulation, protein kinase-mediated phosphorylation is a ubiquitous signaling mechanism in eukaryotic cells [22]. Therefore, exploring the kinome can be an effective approach to understanding signaling pathway regulation. In this study, we utilized the conversion of hEPSCs to human naïve pluripotent stem cells as a model to identify novel signaling pathways that regulate human naïve pluripotency and totipotency. To achieve this, we conducted a CRISPR/Cas9-based kinome knockout screen. We validated our findings using small molecules and identified that inhibiting ErbB signaling promoted the induction of human naïve pluripotency and totipotency *in vitro*.

## Results

### A CRISPR/Cas9-based kinome knockout screen identified kinases that promoted hEPSC conversion to naïve pluripotent stem cells

**Figure 1.**
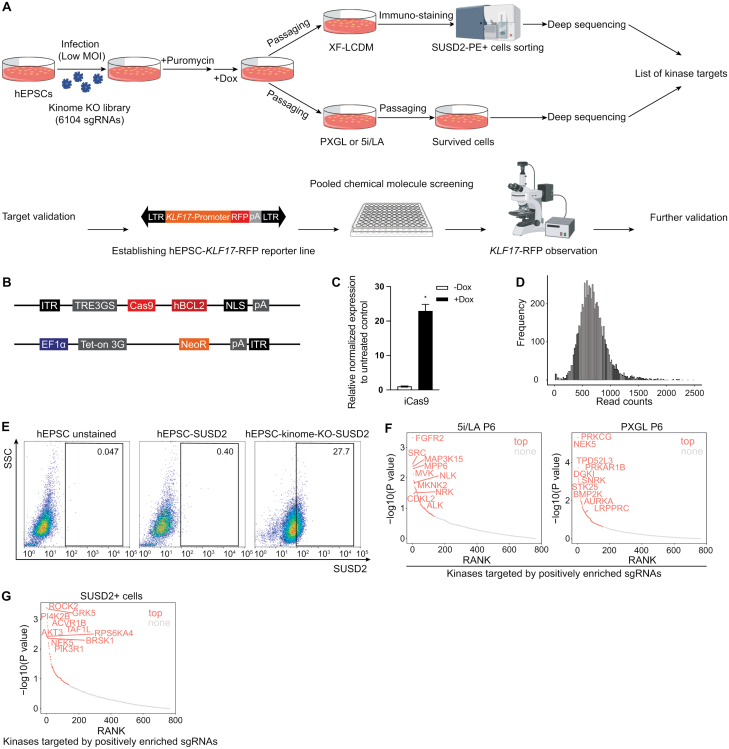
**CRISPR kinome KO screen identified novel targets that increase human EPS cells’ competence of acquiring authentic human naïve state.**(A) Schematics of CRISPR knockout screen with kinome library under naïve or extended pluripotent conditions. (B) Plasmid design of iCas9 expression with BCL2 and Neomycin resistance. iCas9, induced Cas9. (C) Quantitative polymerase chain reaction (q-PCR) analysis of the expression of iCas9 in hEPSC-iCas9-hBCL2-Neo cell line upon Dox treatment. *N* = 2 biological replicates. Error bar indicates standard error of the mean (SEM). (D) The distribution diagram of the initial sgRNA library read counts frequency. (E) Representative flow cytometry sorting of SUSD2 positive cells during kinome KO screen under XF-LCDM condition. Control hEPSCs without SUSD2-phycoerythin (PE) staining (left), control hEPSCs with SUSD2-PE staining (middle), and kinome KO EPSCs with SUSD2-PE staining (right) are presented. The percentage of SUSD2-PE positive cells is shown. This experiment was repeated at least three times. (F) Kinases targeted by positively enriched sgRNAs in cells at passage 6 under naïve pluripotent condition 5i/LA (left) and PXGL (right). Top enriched genes and commonly enriched genes under two conditions are shown. (G) Kinases targeted by positively enriched sgRNAs in sorted SUSD2 positve cells at passage 3 under XF-LCDM condition. Top enriched genes are shown.

To identify kinase candidates that negatively regulate human naïve pluripotency, we conducted a CRISPR knockout screening using hEPSCs cultured under the xeno-free condition termed XF-LCDM [17]. To achieve this, we first integrated a doxycycline (Dox)-inducible Cas9 cassette into hEPSCs (iCas9-hEPSCs) (Fig. 1B and 1C). To minimize the potential toxic effects of CAS9 induction on human pluripotent cells, we over-expressed BCL2 in the Cas9-integrated cell line (Fig. 1B), as previous studies reported that Cas9 expression can induce TRP53-mediated toxic response in human pluripotent cells [23]. To construct a sgRNA library targeting all 763 human kinase genes, we selected corresponding sgRNAs from the Brunello human genome KO library [24], and a total of 6204 sgRNAs were included in the kinome sgRNA library (Table S1). This pooled lentiviral sgRNA library was transduced into the iCas9-hEPSCs, followed by puromycin selection to eliminate cells without sgRNA integration. Deep-sequencing analysis of the frequency distribution of sgRNAs in the transduced cells revealed that the majority of sgRNA read counts varied between 500 and 1000 counts (Fig. 1D). Furthermore, the percentages of mapped sgRNAs in the transduced cells were more than 50%, and the Gini index was low (Gini index = 0.03) (Table S2), indicating a well-covered and diverse sgRNA library in the transduced cells.

Next, we employed two strategies to perform the CRISPR/Cas9-based screens. The first one was based on switching the culture medium from XF-LCDM to PXGL or 5i/LA, respectively (Fig. 1A), which were reported to promote the transition of primed human pluripotent cells to the naïve pluripotent state thus eliminating non-naïve cells after passaging in our screen [13, 25]. Cells were collected at passage 3 and passage 6, and genomic DNA samples were harvested for deep sequencing. Quality control analyses showed well coverage and diversity of sgRNAs in the collected samples from the first screen (Fig. S1A). The second one was based on fluorescence-activated cell sorting (FACS) of sgRNA-transduced cells expressing SUSD2, a representative surface marker of human naïve pluripotent stem cells [26] (Fig. 1A). This strategy did not rely on culturing the sgRNA-transduced cells in the PXGL and 5i/LA conditions, but the XF-LCDM condition was employed during the whole screening process. As a result, the second strategy was favorable for identifying kinase whose knockout led to a more direct transition from the hEPSC state to the naïve pluripotent state. Notably, the percentage of SUSD2-positive cells in the transduced cells was significantly higher than that in the initial iCas9-hEPSCs (Fig. 1E), suggesting that knockout of certain human kinases promoted the conversion of hEPSCs to the naïve state. After sorting, SUSD2-positive cells and the initial transduced cells were collected for further deep sequencing.

Next, we processed the sequencing data from the two screens by comparing gRNA counts between the experimental and control groups using the MAGeCK program [27] (Table S2). Using *P* value < 0.05 as the threshold, sgRNAs that target 279 kinase genes were identified from the first screen (Table S2), which were significantly underrepresented or overrepresented in the cells cultured under the PXGL or 5i/LA condition compared with cells under the XF-LCDM condition before passasing (Table S2). Using the same threshold, sgRNAs that target 78 kinase genes were found to be significantly underrepresented or overrepresented in the SUSD2 positive cells by the second screen (Table S2). We further ranked the sgRNA-target kinases by their enrichment. Because the efficiency and specificity of sgRNA targeting varied among different sgRNAs may lead to noise and bias in CRISPR screens [28], we did not only analyze kinases targeted by sgRNAs that were statistically enriched in the experimental groups (*P* value < 0.05). Instead, we chose the top 100 (13.11% of the 763 screened kinases) enriched genes identified by the MAGeCK program from the first or second screening data, respectively, for further analysis (Table S2). Among the top 100 enriched sgRNA-target kinase genes from the first screen, we identified several commonly enriched kinases that were shared by cells from the PXGL or 5i/LA condition, such as *CDK13*, *BMP2K*, and *MEX3B* (Figs. 1F and S1B; Table S2). The top 100 ranked sgRNA-target kinase genes enriched in the SUSD2 positive cells under the XF-LCDM condition were also identified, such as *ROCK2*, *GRK5*, and *AKT3* (Fig. 1G; Table S2).

### Identification kinases and related signaling pathways that regulate human naïve pluripotency

To validate the effectiveness of our screening for kinases that regulate human naïve pluripotency, we first analyzed whether known kinases that regulate human naïve pluripotency could be identified in our screening. Indeed, sgRNAs targeting several reported kinases [12, 13], such as SRC, PKC, and MEK-ERK, and their upstream and downstream genes along the pathways, were enriched in our sequencing data in both screens (Fig. S1C and S1D).

**Figure 2.**
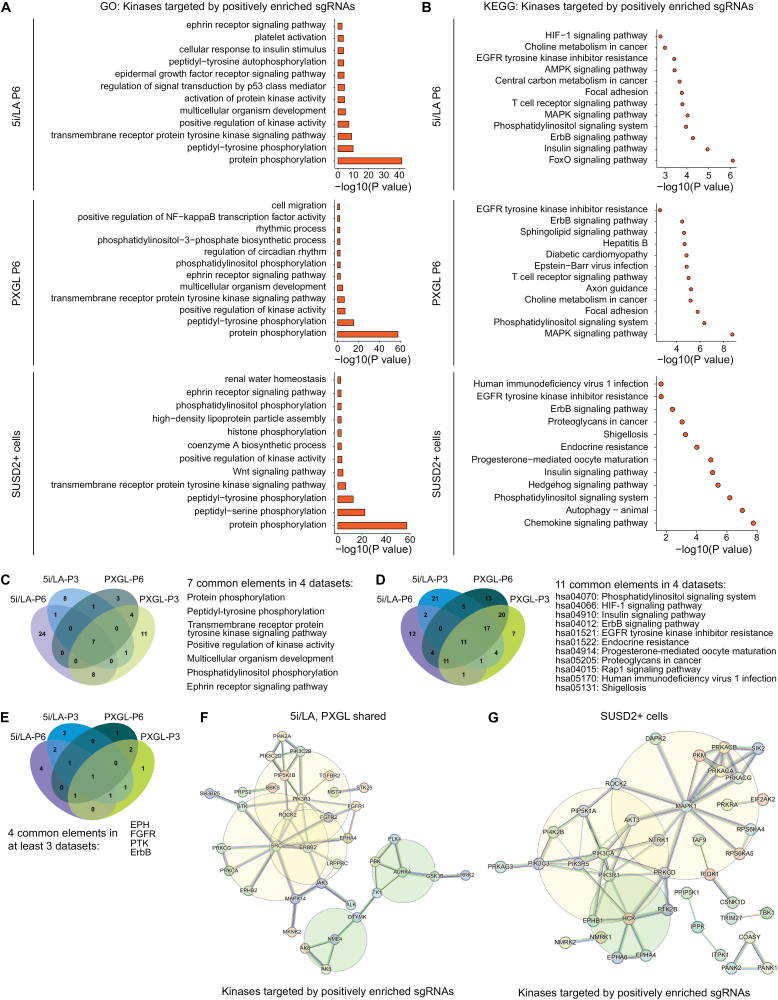
**Analysis of enriched kinases that regulate human naïve pluripotency induction and maintenance.** (A) GO analysis of kinases targeted by positively enriched sgRNAs in cells under 5i/LA (passage 6) (top), PXGL (passage 6) (middle) and sorted SUSD2 positive cells at passage 3 under XF-LCDM condition (bottom). (B) KEGG analysis of kinases targeted by positively enriched sgRNAs in cells under 5i/LA (passage 6) (top), PXGL (passage 6) (middle) and sorted SUSD2 positive cells at passage 3 under XF-LCDM condition (bottom). (C) Venn diagram of enriched GO biological processes terms at passage 3 and passage 6 under PXGL and 5i/LA conditions. Commonly enriched terms are listed. (D) Venn diagram of enriched KEGG signalling pathways at passage 3 and passage 6 under PXGL and 5i/LA conditions. Commonly enriched pathways are listed. (E) Venn diagram of enriched transmembrane receptor protein tyrosine kinase families at passage 3 and passage 6 under PXGL and 5i/LA conditions. Commonly enriched kinase families are listed. (F) PPI analysis of kinases targeted by positively enriched sgRNAs in cells under PXGL and 5i/LA conditions from the first screen. Color of lines between nodes indicated evidence types. Light blue, from curated database, purple, experimentally determined, deep green, gene neighborhood, red, gene fusions, deep blue, gene co-occurrence. Interaction score = 0.700. (G) PPI analysis of kinases targeted by positively enriched sgRNAs in sorted SUSD2 positve cells under XF-LCDM condition from the second screen. Color of lines between nodes indicated evidence types. Interaction score = 0.700.

To explore the biological processes and signaling pathways involved in the induction and maintenance of human naïve pluripotency, we further performed Gene Ontology (GO) and Kyoto Encyclopedia of Genes and Genomes (KEGG) analysis on the kinases that were targeted by sgRNAs positively enriched in the experimental groups (Figs. 2A–D, S1E and S1F). Multiple enriched signaling pathways were identified from the first screen, such as ephrin receptor, ErbB, VEGF, Insulin, FoxO and MAPK signaling pathways (Figs. 2A–D, S1E and S1F). We also identified the common enriched kinase families from the first screening, which included EPH, FGFR, PTK, and ErbB families (Fig. 2E). Similar analysis was performed using the kinases targeted by positively enriched sgRNAs from the second screen, which revealed additional signaling pathways and biological processes regulated by these kinases, such as Wnt signaling, chemokine signaling pathway, autophagy, and hedgehog signaling (Fig. 2A and 2B). Notably, by integrating the results of GO and KEGG analysis from the first and second screens, several common terms emerged, such as insulin, ErbB and ephrin receptor signaling pathways (Fig. S1G and S1H).

To investigate the interactions of these kinases targeted by positively enriched sgRNAs in the experimental groups, we conducted a protein–protein interaction (PPI) network analysis using the STRING tool [29]. We first selected the top 100 enriched kinases from each dataset of the first screen and excluded kinases that appeared only once. The remaining kinases were subjected to PPI analysis, with parameters set to display only strong connections between them. The resulting PPI network revealed that a total of 32 kinases were mapped to 2 major networks. Noteworthy nodes in the larger network included *SRC*, *PIK3R3*, and *ERBB2* (Fig. 2F), whereas those in the smaller network included *NME4* and *AURKA* (Fig. 2F). Similar PPI analysis was performed using the top 100 enriched kinases from the second screen, which showed a major network regulated by MAPK and PI3K signaling pathways (Fig. 2G).

To further reveal kinases involved in regulating human naïve pluripotency, we also analyzed kinases targeted by sgRNAs that were absent or underrepresented in the experimental groups (Fig. S2A; Table S2). In addition to protein phosphorylation, GO analysis revealed that these kinases were involved in other biological processes such as cell cycle regulation, cellular senescence, and lipid phosphorylation (Fig. S2B). Moreover, KEGG analysis showed that several other signaling pathways, such as GnRH signaling, progesterone-mediated oocyte maturation and VEGF signaling (Fig. S2C), were affected. PPI analysis further revealed that the major network of these kinases was affected by PI3K-AKT, PKC, and AMPK signaling pathways (Fig. S2D and S2E).

### Chemical screening verified that inhibition of ErbB signaling promoted the transition of hEPSCs towards naïve pluripotent and totipotent states

**Figure 3.**
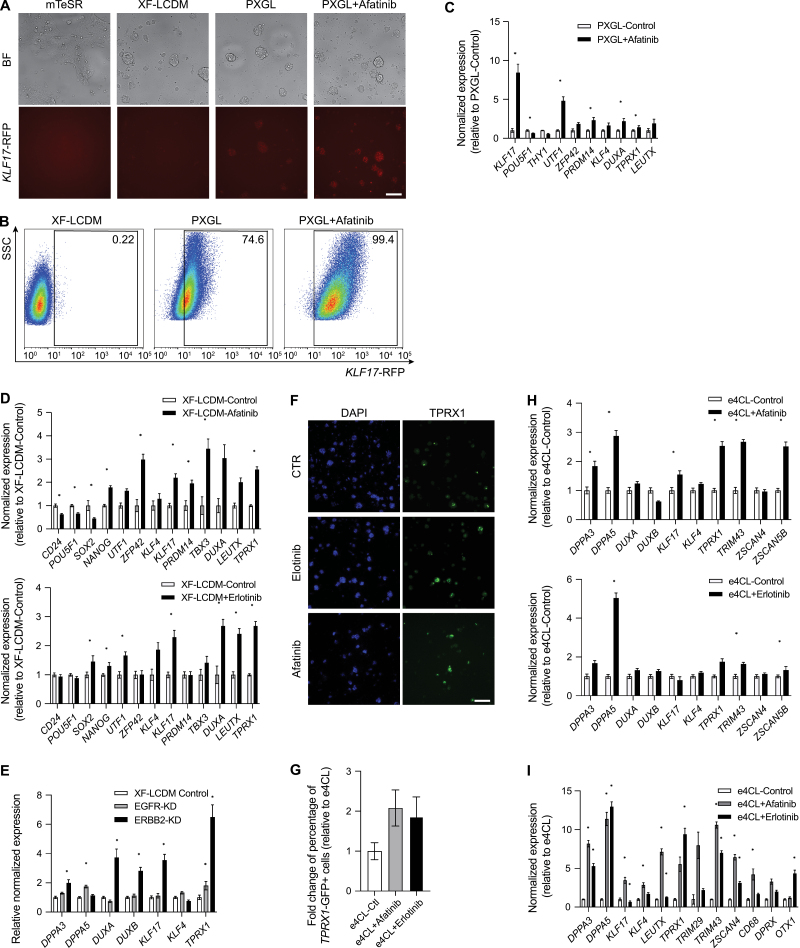
**Chemical validation identified ErbB family inhibitor promotes the transition of hEPSCs toward naïve pluripotency and totipotency.**(A) Representative fluorescent images of *KLF17*-RFP reporter under the mTeSR, XF-LCDM, PXGL, and PXGL with Afatinib conditions. BF, Bright field. Scale bar, 200 μm. This experiment was repeated at least three times. (B) Representative flow cytometry analysis of the percentages of *KLF17*-RFP positive cells under the XF-LCDM, PXGL, and PXGL with Afatinib conditions. This experiment was repeated at least three times. (C) q-PCR analysis of the expression of *KLF17* and marker genes upon Afatinib treatment under the PXGL condition. *N* = 2 biological replicates. Error bar indicates SEM. (D) q-PCR analysis of the expression of naïve pluripotency, primed pluripotency and totipotency marker genes upon Afatinib (upper panel) and Erlotinib (lower panel) treatment under the XF-LCDM condition. *N* = 2 biological replicates. Error bar indicates SEM. (E) q-PCR analysis of the expression of naïve pluripotency and totipotency marker genes upon *EGFR* and *ERBB2* knockdown under the XF-LCDM condition. *N* = 2 biological replicates. Error bar indicates SEM. (F) Resentative immuno-fluorescent images of TPRX1 expression and DAPI upon Afatinib and Erlotinib treatment under the e4CL condition. Scale bar, 200 μm. This experiment was repeated at least three times. (G) Fold change of percentage of *TPRX1*-GFP+ cells relative to e4CL control upon Afatinib and Erlotinib treatment. *N* = 2 biological replicates. Error bar indicates range. (H) Q-PCR analysis of the expression of totipotency marker genes upon Afatinib and Erlotinib treatment under the e4CL condition. *N* = 2 biological replicates. Error bar indicates SEM. (I) Q-PCR analysis of the expression of totipotency marker genes in *TPRX1*-GFP+ sorted cells under e4CL condition and upon Afatinib and Erlotinib treatment under the e4CL condition. *N* = 2 biological replicates. Error bar indicates SEM.

Next, we aimed to functionally verify the roles of newly identified kinases in regulating human naïve pluripotency, and performed a chemical screening as small molecules can regulate the activity of kinases within the same kinase family, making the validation process more efficient. To achieve this, we constructed a small molecule inhibitor pool containing 82 small molecules, which targeted approximately 70% of the kinase families that were targeted by positively enriched sgRNAs (*P* value < 0.05) in the experimental groups from the first and second screens (Fig. 1A; Table S3). As the positive controls, small molecules targeting known kinase regulating human naïve pluripotency were also included, such as SRC and PKC inhibitors. To verify the functional effect of small molecules, we established a *KLF17*-mScarlet reporter cell line (Fig. 1A). *KLF17* is a well-known marker that distinguishes primed pluripotency from naïve pluripotency in humans [30, 31]. Moreover, this gene is highly expressed in human totipotent embryos and *in vitro* induced human 8C-like cells [19, 20], making it suitable for identifying *KLF17* positive totipotent-like cells. Consistent with its roles in marking cells from early preimplantation stages, no significant fluorescence of the *KLF17* reporter was observed in cells cultured in the XF-LCDM medium or mTeSR1 medium (Fig. 3A).

We evaluated the effect of individual small molecules during the conversion of hEPSCs to human naïve pluripotent stem cells using the PXGL condition. Seven small molecules caused severe cell death in the chemical screen (Table S3), the concentration of which may need further titration in the future study. In addition, we observed six small molecules that enhanced the fluorescence of *KLF17* reporter positive cells (Table S3). Notably, the ErbB family inhibitor Afatinib significantly increased the percentage and strength of *KLF17* reporter expression (Fig. 3A and 3B), suggesting that it can promote the conversion of hEPSCs into an earlier state. We further analyzed the molecular characteristics of Afatinib-treated cells using qPCR analysis. Under both PXGL and XF-LCDM conditions, the expressions of multiple naïve pluripotent markers (i.e. *KLF17*, *ZFP42*, *UTF1*) were significantly upregulated whereas those of primed pluripotent markers (i.e. *THY1*, *CD24*) were downregulated (Fig. 3C and 3D), suggesting the promotion of hEPSCs toward the naïve pluripontent state by Afatinib treatment. Consistent with these results, ErbB-related upstream or downstream genes, and ErbB family co-evolved genes were repetitively enriched in the first and second screens (Fig. S3A).

Because *KLF17* is also highly expressed in totipotent cells, we also analyzed whether Afatinib could promote hEPSCs acquiring totipotent features by qPCR analysis. Importantly, we found that the expression of multiple totipotent markers, such as *DUXA*, *LEUTX*, and *TPRX1*, was elevated after adding Afatinib under the PXGL condition (Fig. 3C). Moreover, a similar effect was observed under the XF-LCDM condition (Fig. 3D), and knockdown of *EGFR* or *ERBB2* also led to upregulation of totipotent marker expression in the cells cultured under the XF-LCDM condtion (Fig. 3E). To further analyze the effects of Afatinib on promoting human totipotency, we tested it under the e4CL condition that was reported to promote inducing human totipotency [20]. TPRX1 expression was further enhanced when Afatinib was added to the e4CL condition (Fig. 3F), and the percentages of TPRX1 positive cells were also increased by its treatment (Fig. 3G). Moreover, qPCR analysis showed an upregulation of totipotent marker genes in the e4CL plus Afatinib condition (Fig. 3H). In addition, Afatinib also enhanced the expression of multiple totipotent marker genes in the purified TPRX1 positive cells when compared to the e4CL control (Fig. 3I), which also supported the role of Afatinib in enhancing totipotent features. Besides Afatinib, we also tested Erlotinib, an inhibitor of EGFR signaling that belongs to the ErbB family, and observed similar effects of promoting totipotent marker expression (Fig. 3F-I). Taken together, our small-molecule-pooled screen validated our candidate genes and identified that ERBB family inhibition promoted the transition of hEPSCs towards naïve pluripotent and totipotent states.

### Transcriptomic analysis of Afatinib-induced totipotent features in human pluripotent cells

Because Afatinib is a potent ErbB family inhibitor which can simultaneously inhibit EGFR, ERBB2, and ERBB4 [32], we sought to analyze the expressions of ErbB signaling-related gene members during early human preimplantation development. By re-analyzing data from single-cell RNA-sequencing and Ribo-sequencing of human early preimplantation embryos [33, 34], we found that the expressions of ErbB receptors *EGFR* and *ERBB2* were relatively low or undetectable in 8C embryos when compared to that in human primed pluripotent stem cells (Fig. S3B). *ERBB4* showed relatively higher expression at embryonic stages before the 8C embryo stage, which gradually reduced to an undetectable level after the 8C embryo stage (Fig. S3B). Additionally, the majority of kinases involved in the ErbB signaling pathway were also lowly expressed at the 8C embryo stage (Fig. S3C), suggesting the activity of ErbB signaling was suppressed in the totipotent 8C human embryos.

**Figure 4.**
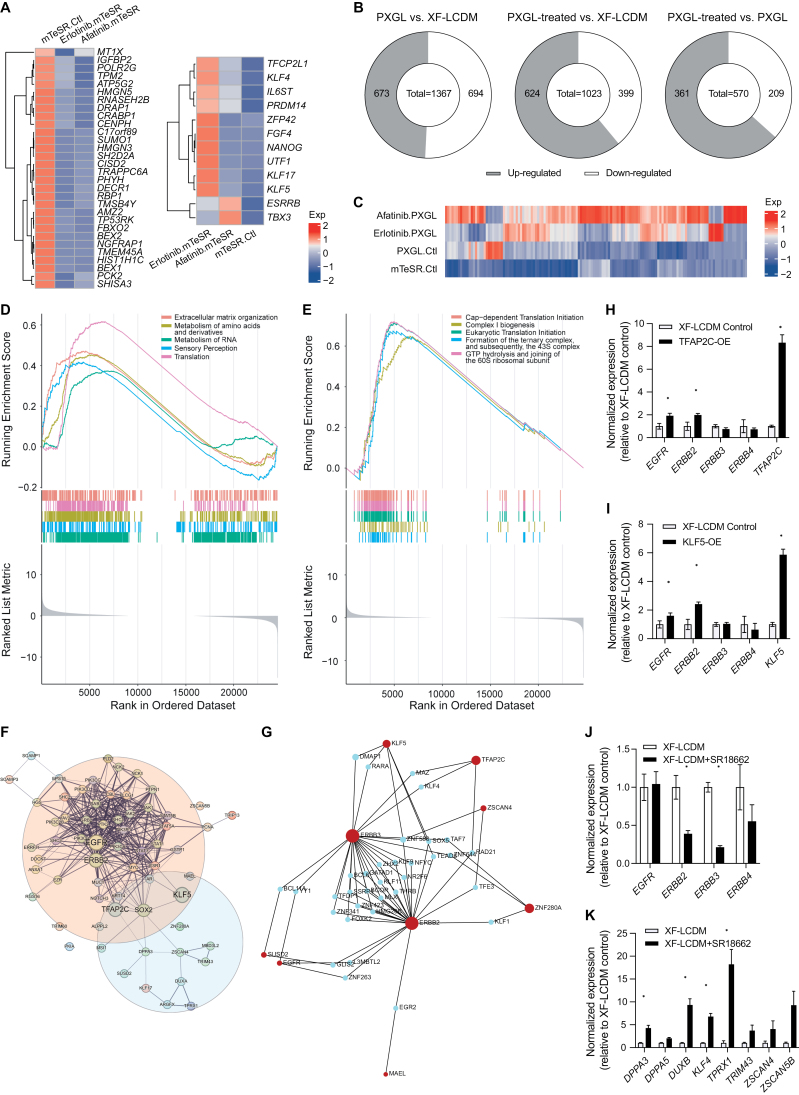
**Transcriptomic analysis of ErbB family inhibitors induced naïve pluripotency and totipotent features.**(A) Heatmap analysis of significantly down-regulated primed pluripotency marker genes (left) and up-regulated naïve pluripotent marker genes (right) upon Afatinib and Erlotinib treatment under the mTeSR condition. (B) Pie chart showing number of up-regulated and down-regulated differentially expressed pan-early embryonic genes under different conditions. PXGL treated, differentially expressed genes shared by PXGL supplemented with Afatinib and that with Erlotinib. (C) Heatmap analysis of totipotency-specific genes under mTeSR, PXGL and PXGL treated with Afatinib and Erlotinib. (D) GSEA enrichment result upon Afatinib treatment under PXGL condition. (E) GSEA enrichment result upon Afatinib treatment under PXGL condition. (F) PPI analysis network showing protein–protein interactions between ErbB family along with their downstream genes and 8CLC hub genes. Interaction score = 0.4. Line thickness indicates the strength of data support. (G) Predicted TF–gene interaction network between ErbB family and 8CLC hub genes. Factor and gene target data derived from the ENCODE ChIP-seq data. Only peak intensity signal < 500 and the predicted regulatory potential score < 1 is used (using BETA minus algorithm). (H) Q-PCR analysis of the expression of ERBB famlily genes and *TFAP2C* upon *TFAP2C* transient over-expression under the XF-LCDM condition. *N* = 2 biological replicates. Error bar indicates SEM. (I) q-PCR analysis of the expression of ERBB family genes and *KLF5* upon *KLF5* transient over-expression under the XF-LCDM condition. *N* = 2 biological replicates. Error bar indicates SEM. (J) q-PCR analysis of the expression of ERBB family genes upon SR18662 treatment under the XF-LCDM condition. *N* = 2 biological replicates. Error bar indicates SEM. (K) q-PCR analysis of the expression of totipotency marker genes upon SR18662 treatment under the XF-LCDM condition. *N* = 2 biological replicates. Error bar indicates SEM.

To further investigate the effect of ErbB inhibition on inducing totipotent features, we analyzed the transcriptomic changes induced by ErbB or EGFR inhibitors under different culturing conditions, including mTeSR1, XF-LCDM, and PXGL (Table S4). In mTeSR1 medium, both Afatinib and Erlotinib downregulated the expression of representative primed pluripotency markers (Fig. 4A). Notably, Erlotinib was found to be more effective than Afatinib in promoting the expression of naïve pluripotency markers under the mTeSR1 condition (Fig. 4A). We then focused on analyzing whether ErbB or EGFR inhibitors promoted the transition of pluripotent states toward an early embryonic state. Using the transcriptomic data set generated by Yan et al. [33], we identified gene clusters that were upregulated from the oocyte to the morula stage during the preimplantation stages in humans (Fig. S3C), which included 6377 genes and were defined as pan-early embryonic genes. Additionally, we also identified gene clusters that were enriched in primed pluripotent stem cells (Fig. S3D). By comparing to the non-treated controls, we found that multiple pan-early embryonic genes were upregulated in Afatinib or Erlotinib-treated groups under the mTeSR1, XF-LCDM, or PXGL conditions, respectively (Fig. S4A). Furthermore, genes enriched in primed pluripotent stem cells were downregulated upon the treatment with Afatinib or Erlotinib (Fig. S4A). These results suggest that chemical inhibition of ErbB signaling suppresses transcriptomic features of primed pluripotency and promotes the induction of early embryonic transcriptomic features. On the other hand, we also noticed significant transcriptomic differences between Afatinib and Erlotinib treated samples, which may be caused by the differences ininhibiting ErbB-related receptors and related genes between Afatinib and Erlotinib. In addition, it is also possible that off-target effects of these two small molecules may contribute to these differences.

Because the PXGL condition has been shown to support the presence of a totipotent-like cell subpopulation in human naïve pluripotent cell cultures [18], we focused on analyzing the transcriptomic changes that occur upon the addition of Afatinib or Erlotinib in the PXGL condition. To this end, we first identified differentially expressed genes between cells cultured under the XF-LCDM condition and those under the Afatinib+PXGL, Erlotinib+PXGL, and PXGL conditions respectively. Among these different genes, we focused on analyzing genes that were also pan-early embryonic genes. Among the differentially expressed genes between the PXGL and XF-LCDM condition, 1367 genes were identified as pan-early embryonic genes, 49.2% of which were upregulated in the PXGL condition when compared to the XF-LCDM condition (Fig. 4B). Using the XF-LCDM condition as the control, 1023 differentially expressed genes were shared by Afatinib+PXGL and Erlotinib+PXGL conditions and were identified as pan-early embryonic genes, 60.9% of which were upregulated when compared to the XF-LCDM condition (Fig. 4B). Similarly, when using the PXGL condition as the control, 63.3% of the pan-early embryonic genes from the differentially expressed genes that were shared by Afatinib+PXGL and Erlotinib+PXGL conditions were upregulated when compared to the PXGL condition (Fig. 4B). To further analyze the transcriptional changes of totipotent 8C embryonic features upon ErbB inhibition, we integrated reported totipotent 8C marker genes and defined an 8C totipotent signature that contained 141 genes [19, 20, 35] (Fig. S3E; Table S5). Notably, the majority of the 8C totipotent signature was upregulated by Afatinib or Erlotinib treatment under the PXGL condition (Fig. 4C). Furthermore, GSEA analysis showed that both Afatinib and Erlotinib treatment led to the enrichment of genes that regulate the protein translation process (Fig. 4D), which plays an important role in regulating early human embryonic development [36]. These data suggest that ErbB inhibition promotes the induction of totipotent 8C signatures from the human naïve pluripotent state.

To understand the potential mechanisms underlying the enhanced totipotency induction by ErbB inhibition, we explored the biological processes and signaling pathways that were affected by ErbB inhibition (Fig. S4B and S4C). Using genes that were differentially expressed between Afatinib or Erlotinib treated groups and the controlled PXGL group, we performed GO and KEGG analysis (Fig. S4B and S4C). Notably, the top GO terms included protein translation and regulation of transcription (Fig. S4B), which were shown to be major pathways during the human embryonic genome activation process [36]. Consistent with this result, KEGG analysis showed that signaling pathways relating to ribosome regulation were mostly enriched (Fig. S4C). Additionally, oxidative phosphorylation, which was related to mitochondria function, was also enriched (Fig. S4C).

To further explore the interaction between ErbB signaling and the core gene regulatory network (GRN) of human totipotency, we analyzed the protein interaction between the hub genes from the GRN of human 8C-like cells (8CLC) and ErbB family kinases as well as their downstream targets [19]. Notably, we observed that the major node genes that connected ErbB signaling to the totipotent GRN in human 8CLC included *KLF5* and *TFAP2C* (Fig. 4F). This observation was consistent with the prediction of TF–gene interaction network between ErbB family members and 8C-specific genes, which also showed that *KLF5* and *TFAP2C* interacted with the major ErbB family members (Fig. 4G). Interestingly, we found that transient overexpression of *KLF5* or *TFAP2C* in hEPSCs under the XF-LCDM condition upregulated *EGFR* and *ERBB2* (Fig. 4H and 4I). Moreover, inhibition of KLF5 function by applying a KLF5 inhibitor SR18662 not only led to downregulation of *ERBB2* and *ERBB3* (Fig. 4J) but also upregulated the expressions of totipotent marker genes (Fig. 4K). Collectively, these data indicate that ErbB signaling plays an important role in regulating key biological processes involved in early human embryo development and interacts with the core GRN of human totipotency.

## Discussion

**Figure 5.**
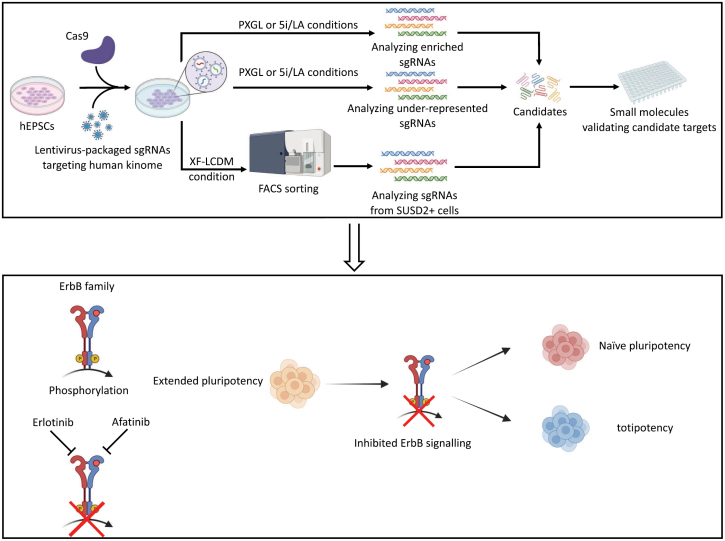
Graphic summary. We performed a comprehensive CRISPR/Cas9-based kinome knockout screen with different strategies on hEPSCs and identified new kinases and related signaling pathways that regulate human naïve pluripotency. Chemical validation of these newly identified kinases revealed that inhibition of ErbB signaling not only promoted the induction of human naïve pluripotency but also human totipotency *in vitro*.

In this study, using the conversion of hEPSCs to human naïve pluripotent stem cells as a model, we performed a comprehensive CRISPR/Cas9-based kinome knockout screen and identified new kinases and related signaling pathways that regulate human naïve pluripotency. More importantly, chemical validation of these newly identified kinases revealed that inhibition of ErbB signaling not only promoted induction of human naïve pluripotency but also human totipotency *in vitro* (Fig. 5). These findings are favorable for deepening the understanding of mechanisms of human naïve pluripotency and totipotency regulation.

Our screen uncovered new kinases that underpin the core signaling regulatory network of human naïve pluripotency. Notably, 94.5% of kinases (315/333) identified in our study were not discovered in a recent genome-wide CRISPR/Cas9-based screening that identified regulators of human primed to naïve pluripotent stem cell reprogramming [37] (Table S2). Compared to the genome-wide CRISPR/Cas9-based screening study that used a flow cytometry strategy [37], our study employed a more focused sgRNA library and combined both viability screens and flow cytometry strategy to perform the pooled screen (Fig. 1A). Therefore, the reduction in library size could allow the generation of cleaner data and the complementary use of viability screens and flow cytometry strategy may facilitate efficient identification of functional targets [38]. On the other hand, we also noticed that the overlap of genes identified from the two screens was not high, as only 24 enriched genes were identified from both two screens. This may be explained by the different strategies of enriching target in the two screens, because extended culturing cells under the naïve conditions in the first screen may favor the selection of genes that promote the survival and proliferation of naïve pluripotent cells, and purifying naïve cells by flow cytometry may facilitate identifying genes that promote direct conversion to the naïve state. Despite the differences in the enriched kinase genes, we identified several common signaling pathways and biological processes from the two screens (Fig. S1G and S1H), suggesting both screens enabled the discovery of kinase genes regulating human naïve pluripotency.

Notably, analysis of the PPI network of the identified kinases suggested that the core regulatory signaling pathways of human naïve pluripotency included not only MAPK signaling that governs mouse naïve pluripotency regulation [7] but also SRC, PI3K-AKT, PKC, and AMPK signaling (Figs. 2F and 2G, S2D and S2E). Moreover, bioinformatic analysis of these newly identified kinases revealed several novel signaling pathways that were involved in regulating human naïve pluripotency, such as ephrin receptor, ErbB, VEGF, insulin, and FoxO signaling pathways (Fig. 2A and 2B, S1B and S1C), which were not reported in previous studies that stabilized human naïve pluripotent stem cells by signaling pathway regulation [12, 13, 39]. These results provide a foundation for future explorations of the newly identified signaling pathways that potentially regulate human naïve pluripotency and would have important implications for the derivation of human stem cells from early preimplantation developmental stages.

More importantly, our study has established a new role for ErbB signaling in the regulation of human totipotency *in vitro*. While ErbB signaling has been extensively studied in cancer biology [40], its involvement in the regulation of totipotency and early preimplantation embryonic development in humans has remained largely unknown. Our data showed that the ErbB inhibitor Afatinib effectively promoted the induction of typical molecular characteristics of human totipotency under different culturing conditions for human pluripotent cells (Figs. 3C, 3D and S1A), as well as the e4CL condition for inducing human totipotent-like cells *in vitro* (Fig. 3F-I). In support of these findings, independent analysis of expressions of ErbB family members in the early human preimplantation embryos suggests that the activity of ErbB signaling is suppressed in totipotent 8C human embryos (Fig. S3B and S3C). Moreover, GO and KEGG analysis revealed that ErbB inhibition significantly affected protein translation, regulation of transcription, and oxidative phosphorylation (Fig. S4B and S4C), which are majorly involved in the regulation of the transition from human 4–8 embryos to morulae [36]. These observations indicate that ErbB signaling plays important roles not only in the induction of human totipotency *in vitro* but also actively participates in early human preimplantation development. Interestingly, both TF–gene interaction and PPI analysis suggest that ErbB signaling interacts with *TFAP2C* and *KLF5* (Fig. 4F and 4G), which participate in the regulatory network of human 8C-like cells and have been reported to be upstream activators of ErbB signaling [19, 41, 42]. Considering that these two TFs are implicated in directing extraembryonic trophectodermal fate in early preimplantation blastomeres or embryonic stem cells [43, 44], it is possible that their downstream ErbB signaling could be an important driver that regulate the transition of human totipotent state to the non-totipotent state. Future studies of the mechanisms of ErbB signaling in regulating human totipotency and early preimplantation development would be beneficial for understanding cell fate determination in early human development.

In summary, our study has revealed new kinases and signaling pathways involved in the regulation of human naïve pluripotency. The screening datasets generated in this study provide a valuable resource for exploring novel signaling pathways and regulators that control human naïve pluripotency and totipotency in the future. Furthermore, our study has also highlighted the ErbB signaling pathway as a new regulator that induces human totipotency *in vitro*, which may play an active role in early human preimplantation embryo development. These findings provide significant insights into the mechanisms governing human naïve pluripotency and totipotency regulation and will advance the optimization of *in vitro* culturing conditions for human naïve pluripotent stem cells and human totipotent stem cells in the future.

### Research limitations

Although we identified multiple new kinases and related signaling pathways involved in the regulation of human naïve pluripotency, the functional roles of these newly identified targets in regulating human naïve pluripotency have not been extensively studied. In addition, a more specific CRISPR/Cas9 screening strategy for identifying regulators of human totipotency is required to be developed. Future studies are needed to clarify the mechanisms of human naïve pluripotency regulation by these newly identified kinases, as well as to systematically identify novel regulators of human totipotency *in vitro*.

### Methods

#### Cell culture

Human EPSCs were cultured in a xeno-free LCDM medium on Matrigel [17]. H1-ESCs were cultured in mTeSR1 medium (Stem cell technology, 85850) on Matrigel. Naive human PSCs were propagated in PGXL or 5i/LA medium on Matrigel [13, 25]. 8CLCs were first cultured in 4CL medium [20]. Three passages later the medium was switched to e4CL medium [20].

#### Plasmid design and construction

For plasmid PB-iCas9-hBCL2-CMV-Rtta-Neo, the Cas9 sequence was obtained from pAAVS1-tet-iCas9-BFP2 vector (addgene#125519). The human BCL2 (hBCL2) sequence (NM_000633.3) and synthesized by Genscript. The backbone vector was originated from PB-TRE-dCas9-VPR (addgene#63800), in which the HygR sequence was replaced by NeoR and dCas9-VPR sequence was replaced by Cas9-P2A-hBCL2 sequence.

For plasmid PB-*KLF17*-mScarlet-NLS-PGK-Hygro, the regulatory sequence started from -2 kb to transcription start site (TSS) of human *KLF17* gene was synthesized by Genscript. The mScarlet and PGK promoter sequences were obtained from addgene#110623 and addgene#31937. The *KLF17* regulatory sequence, mScarlet with an nuclear localization signal (NLS) in C terminal, SV40 polyA, and PGK promoter were amplified with PrimeSTAR Max DNA Polymerase kit (TAKARA) and assembled using NEBuilder HiFi DNA Assembly kit (NEB). The backbone vector originated from addgene#63800, in which the TRE-dCas9-VPR-CMV sequence was replaced by KLF17-mScarlet-NLS-PGK sequence.

#### Cell line establishment

hEPSC-Piggybac-iCas9-hBCL2-Neo cell line was established by nucleofection of PBase and PB-iCas9-hBCL2-CMV-Rtta-Neo plasmids into hEPSCs under XF-LCDM medium. Cells were then cultured in XF-LCDM medium supplemented with 400 μg/mL G418 for two passages. The remaining cells were recovered for one passage and then single clones were picked. hEPSC-Piggybac-*KLF17*-mScarlet-Hygro cell line was established by nucleofection of PBase and plasmid PB-*KLF17*-mScarlet-NLS-PGK-Hygro plasmids into hEPSCs under XF-LCDM medium. Cells were then cultured in XF-LCDM medium supplemented with 100 μg/mL Hygromycin for two passages. The remaining cells were recovered for one passage and then single clones were picked.

#### Kinome-wide CRISPR-Cas9 knockout screening

Human Kinome CRISPR Knockout Library (addgene, #1000000082) with a total of 6204 single guide RNAs (Table S1) were used. Lentivirus of the kinome CRISPR library was produced in HEK293T cells by co-transfection of the sgRNA pooled library together with pMD2.G (addgene#12259) and psPAX2 (addgene#12260) using polyethyleneimine (PEI). Supernatants were collected 48 h and 96 h post-transfection and centrifuged at 7000 rcf to remove cell residue and the lentivirus-containing was concentrated with PEG8000.

The lentiviral kinome library was delivered into hEPSC-Piggybac-iCas9-hBCL2-Neo cell line in XF-LCDM medium with the cell density of 6.8 × 10E7 per T225 flask, in a total of 10 T225 flasks. Two days post-infection 2 µg/ml puromycin were supplemented into the culture medium. After 2 days, the dead cells were washed off with PBS, the remaining cells were trypsinized with 0.05% trypsin and seeded into a new dish with the density of 6.8 × 10E7 per T225 flask in XF-LCDM containing 2 µg/ml puromycin. Cells were purified for another 4 days before screening. After puromycin selection, cells were passaged and equally divided for further screening. 1/3 of the total cells were then switched to culture under PXGL condition, 1/3 were switched to 5i/LA condition. Cells were then collected every three cell passages with the counts more than 6 × 10E6 (minimum average 100 cells per sgRNA) to harvest sgRNAs for analysis. The remaining 1/3 of the total cells were continually cultured under XF-LCDM conditions and collected in three passages. 1/3 of the total cells under XF-LCDM condition were immuno-stained with SUSD2-PE antibody (Miltenyi Biotec, 130-106-399). The stained cells were then sorted with FACS for PE-positive cells.

#### EGFR and ERBB2 knockdown

Lenti-dCas9-KRAB-MeCP2-BSD plasmid (addgene#122205) was packaged with pMD2.G (addgene#12259) and psPAX2 (addgene#12260) using PEI in HEK293T cells. Supernatants were collected 48 h and 96 h post-transfection and centrifuged at 7000 rcf to remove cell residue and the lentivirus-containing was concentrated with PEG8000. gRNAs targeting *EGFR* or *ERBB2* cloned to a Piggybac vector with puromycin resistance were transduced into hEPSCs via nucleofection with PBase plasmid. Cells were cultured under XF-LCDM condition for 1 day and then switched to XF-LCDM supplemented with 2 μg/mL puromycin and purified for two passages. Cells were then infected with dCas9-KRAB-MeCP2-BSD lentivirus and purified with 10 μg/mL BSD for two passages.

#### KLF5 and TFAP2C over-expression

CMV-*KLF5*-Neo (Origene, RC202438) and CMV-*TFAP2C*-Neo (Origene, RC208665) were transduced into hEPSCs via nucleofection. Cells were cultured under XF-LCDM condition for 1 day and then switched to XF-LCDM supplemented with 400 μg/mL G418 for 3 days.

#### Flow cytometry

hEPSCs were dissociated with 0.05% trypsin. Cell pellets were washed in 5 mL PBS twice. The cells were then resuspended in FACS buffer (PBS supplemented with 2% FBS), and incubated with antibody anti-SUSD2-PE (1:100) for 30 min on ice in the dark. Cells were then washed twice with 5 mL PBS pre-incubated on ice, resuspended in 1 mL FACS buffer, and passed through a 0.4 μm cell strainer. Flow cytometry was performed using BD FACSAria SORP and the data were analyzed using Flowjo software.

#### Genome extraction and deep-sequencing library preparation

Genome DNAs were isolated with QIAamp DNA Mini Kit (QIAGEN, 51304). For each Deep-sequencing sample, we performed 100 μL reactions with 5 μg genomic DNA in each reaction using Q5 High Fidelity Master Mix (NEB, M0492S). Primers used for sgRNA library amplification are listed in Table S8. The PCR products were purified using the QIAquick Gel Extraction Kit (QIAGEN, 28704).

#### Whole-kinome CRISPR-KO screen data analysis

The Deep-sequencing samples were sequenced on a NovaSeq 6000 (Illumina). The screening data were analyzed using the Model-based Analysis of Genome-wide CRISPR-Cas9 Knockout (MAGeCK v0.5.9.4) algorithm. Quality control and plotting were performed with MAGeCKFlute. GO and KEGG analysis for identified genes were performed using DAVID bioinformatics resources.

#### Protein–protein interaction analysis

PPI analysis was performed with STRING Database. The list of positively and negatively enriched genes was submitted for mapping. Full STRING network was performed. All interaction sources were activated and the minimum required interaction score was set as 0.700 or 0.400. Disconnected nodes were hidden.

#### TF–gene interaction analysis

TF–gene interaction analysis was performed on NetworkAnalyst with Encode database. TF and gene target data derived from the ENCODE ChIP-seq data. Only peak intensity signal <500 and the predicted regulatory potential score <1 were used (using a BETA minus algorithm). TFs only connected to one node were manually hidden.

#### Chemical validation of candidate targets

Candidate targets were generated from the screening results. hEPSC-*KLF17*-TdTomato reporter cell line was cultured in either XF-LCDM or PXGL medium and seeded to 96-well plate at the concentration of 2 × 10E6 each plate. Chemical small molecules were added into the medium the second day cells were seeded at the concentrations of 1 μM and 2 μM. The observation of fluorescence was 3 days afterward.

#### Immunofluorescence of TPRX1 for cells under e4CL condition

The cells were fixed in 4% paraformaldehyde for 10 min. Fixed cells were permeabilized with PBS plus 0.2% Triton X-100 for 30 min and blocked with 2% BSA for 1 h. The cells were incubated with primary TPRX1 antibody (1:500, Novus Biologicals, NBP1-92524) at 4°C overnight and washed with PBS 3 times. Then the cells were incubated with a secondary antibody for 1 h. The nuclei were stained with DAPI at room temperature for 5 min and washed three times with PBS.

#### Imaging

The imaging was performed in live conditions within culture media for chemical validation and fixed condition within PBS for immunofluorescence. All images were captured at 10× magnification on a Nicon eclipse Ti microscope.

#### qPCR analysis

Isolated RNAs were converted to cDNA using TransScript First-Strand cDNA Synthesis SuperMix (TransGen Biotech, AT311). qPCR analysis was conducted using KAPA SYBR FAST qPCR Kit (KAPA Biosystems, KK4601) with Bio-Rad CFX Real-Time System. The primers used for qPCR analysis are listed in Table S6. The data were analyzed using the ΔΔCt method. Error bars represent the standard error of the mean of the replicates. Asterisk (*) represent statistical significance (*P* value < 0.05).

#### Bulk RNA-sequencing and data analysis

RNA libraries for RNA-seq were prepared using the KAPA Hyper Prep Kits (KK8504). RNA sequencing reads generated from each sample were aligned to the human genome (UCSC h19) with TopHat (v2.1.1). Mapped reads were assembled into genes guided by reference annotation (h19, USCS gene annotation) with Cufflink. Differential gene was conducted by using the Cuffdiff program (*P* value < 0.05). The read counts for each gene were calculated, and the expression values of each gene were normalized using FPKM. Heatmap was generated with R package ComplexHeatmap. Volcano plot was drawn with R package ggplot2. GSEA was performed with R package clusterProfiler.

#### Statistical analysis

Statistical significances were calculated with ONE WAY ANOVA analysis. Asterisk (*) represent statistical significance (*P* value < 0.05).

#### Public data analysis

We used public data GSE36552 and GSE165782 in this article. Clustering was performed with data GSE36552 and processed with Tight clustering algorithm.

#### Research ethics

H1 human ES cell line was purchased from WiCell and all the experiments were performed under relative ethical guidelines of WiCell and ISSCR guidelines of stem cell research.

## Data availability

Bulk-RNA sequencing data are deposited at NCBI’s GEO under the accession number GSE233760.

## Supplementary Material

lnad037_suppl_Supplementary_Figures

lnad037_suppl_Supplementary_Table_S1

lnad037_suppl_Supplementary_Table_S2

lnad037_suppl_Supplementary_Table_S3

lnad037_suppl_Supplementary_Table_S4

lnad037_suppl_Supplementary_Table_S5

lnad037_suppl_Supplementary_Table_S6

lnad037_suppl_Supplementary_Data

## References

[1] Baker CL, Pera MF. Capturing totipotent stem cells. Cell Stem Cell 2018;22:25–34. 29304340 10.1016/j.stem.2017.12.011

[2] Molè MA, Weberling A, Zernicka-Goetz M. Comparative analysis of human and mouse development: from zygote to pre-gastrulation. Curr Top Dev Biol 2020;136:113–38. 31959285 10.1016/bs.ctdb.2019.10.002

[3] Guo G, Stirparo GG, Strawbridge SE et al. Human naive epiblast cells possess unrestricted lineage potential. Cell Stem Cell 2021;28:1040–1056.e6. 33831366 10.1016/j.stem.2021.02.025 PMC8189439

[4] Zou Z, Zhang C, Wang Q et al. Translatome and transcriptome co-profiling reveals a role of TPRXs in human zygotic genome activation. Science 2022;378:abo7923. 36074823 10.1126/science.abo7923

[5] De Iaco A, Planet E, Coluccio A et al. DUX-family transcription factors regulate zygotic genome activation in placental mammals. Nat Genet 2017;49:941–5. 28459456 10.1038/ng.3858 PMC5446900

[6] Whiddon JL, Langford AT, Wong CJ et al. Conservation and innovation in the DUX4-family gene network. Nat Genet 2017;49:935–40. 28459454 10.1038/ng.3846 PMC5446306

[7] Martello G, Smith A. The nature of embryonic stem cells. Annu Rev Cell Dev Biol 2014;30:647–75. 25288119 10.1146/annurev-cellbio-100913-013116

[8] Li M, Belmonte JCI. Deconstructing the pluripotency gene regulatory network. Nat Cell Biol 2018;20:382–92. 29593328 10.1038/s41556-018-0067-6 PMC6620196

[9] Kinoshita M, Barber M, Mansfield W et al. Capture of mouse and human stem cells with features of formative pluripotency. Cell Stem Cell 2021;28:453–71.e8. 33271069 10.1016/j.stem.2020.11.005 PMC7939546

[10] Yu L, Wei Y, Sun HX et al. Derivation of intermediate pluripotent stem cells amenable to primordial germ cell specification. Cell Stem Cell 2021;28:550–567.e12. 33271070 10.1016/j.stem.2020.11.003

[11] Nichols J, Smith A. Naive and primed pluripotent states. Cell Stem Cell 2009;4:487–92. 19497275 10.1016/j.stem.2009.05.015

[12] Takashima Y, Guo G, Loos R et al. Resetting transcription factor control circuitry toward ground-state pluripotency in human. Cell 2014;158:1254–69. 25215486 10.1016/j.cell.2014.08.029 PMC4162745

[13] Theunissen TW, Powell BE, Wang H et al. Systematic identification of culture conditions for induction and maintenance of naive human pluripotency. Cell Stem Cell 2014;15:471–87. 25090446 10.1016/j.stem.2014.07.002 PMC4184977

[14] Theunissen TW, Friedli M, He Y et al. Molecular criteria for defining the naive human pluripotent state. Cell Stem Cell 2016;19:502–15. 27424783 10.1016/j.stem.2016.06.011 PMC5065525

[15] Io S, Kabata M, Iemura Y et al. Capturing human trophoblast development with naive pluripotent stem cells *in vitro*. Cell Stem Cell 2021;28:1023–1039.e13. 33831365 10.1016/j.stem.2021.03.013

[16] Yang Y, Liu B, Xu J et al. Derivation of pluripotent stem cells with in vivo embryonic and extraembryonic potency. Cell 2017;169:243–257.e25. 28388409 10.1016/j.cell.2017.02.005 PMC5679268

[17] Liu B, Chen S, Xu Y et al. Chemically defined and xeno-free culture condition for human extended pluripotent stem cells. Nat Commun 2021;12:3017. 34021145 10.1038/s41467-021-23320-8 PMC8139978

[18] Sozen B, Jorgensen V, Weatherbee BAT et al. Reconstructing aspects of human embryogenesis with pluripotent stem cells. Nat Commun 2021;12:5550. 34548496 10.1038/s41467-021-25853-4 PMC8455697

[19] Taubenschmid-Stowers J, Rostovskaya M, Santos F et al. 8C-like cells capture the human zygotic genome activation program *in vitro*. Cell Stem Cell 2022;29:449–459.e6. 35216671 10.1016/j.stem.2022.01.014 PMC8901440

[20] Mazid MA, Ward C, Luo Z et al. Rolling back human pluripotent stem cells to an eight-cell embryo-like stage. Nature 2022;605:315–24. 35314832 10.1038/s41586-022-04625-0

[21] Weinberger L, Ayyash M, Novershtern N et al. Dynamic stem cell states: naive to primed pluripotency in rodents and humans. Nat Rev Mol Cell Biol 2016;17:155–69. 26860365 10.1038/nrm.2015.28

[22] Johnson SA, Hunter TK. Methods for deciphering the kinome. Nat Methods 2005;2:17–25. 15789031 10.1038/nmeth731

[23] Ihry RJ, Worringer KA, Salick MR et al. p53 inhibits CRISPR-Cas9 engineering in human pluripotent stem cells. Nat Med 2018;24:939–46. 29892062 10.1038/s41591-018-0050-6

[24] Doench JG, Fusi N, Sullender M et al. Optimized sgRNA design to maximize activity and minimize off-target effects of CRISPR-Cas9. Nat Biotechnol 2016;34:184–91. 26780180 10.1038/nbt.3437 PMC4744125

[25] Bredenkamp N, Yang J, Clarke J et al. Wnt inhibition facilitates RNA-mediated reprogramming of human somatic cells to naive pluripotency. Stem Cell Rep 2019;13:1083–98. 10.1016/j.stemcr.2019.10.009 PMC6915845 31708477

[26] Bredenkamp N, Stirparo GG, Nichols J et al. The cell-surface marker sushi containing domain 2 facilitates establishment of human naive pluripotent stem cells. Stem Cell Rep 2019;12:1212–22. 10.1016/j.stemcr.2019.03.014 PMC6565611 31031191

[27] Li W, Xu H, Xiao T et al. MAGeCK enables robust identification of essential genes from genome-scale CRISPR/Cas9 knockout screens. Genome Biol 2014;15:554. 25476604 10.1186/s13059-014-0554-4 PMC4290824

[28] Hsu PD, Scott DA, Weinstein JA et al. DNA targeting specificity of RNA-guided Cas9 nucleases. Nat Biotechnol 2013;31:827–32. 23873081 10.1038/nbt.2647 PMC3969858

[29] Szklarczyk D, Kirsch R, Koutrouli M et al. The STRING database in 2023: protein–protein association networks and functional enrichment analyses for any sequenced genome of interest. Nucleic Acids Res 2023;51:D638–D646.28. 36370105 10.1093/nar/gkac1000 PMC9825434

[30] Lea RA, McCarthy A, Boeing S et al. KLF17 promotes human naïve pluripotency but is not required for its establishment. Development 2021;148:dev199378. 34661235 10.1242/dev.199378 PMC8645209

[31] Wang SH, Hao J, Zhang C et al. KLF17 promotes human naive pluripotency through repressing MAPK3 and ZIC2. Sci China Life Sci 2022;65:1985–97. 35391627 10.1007/s11427-021-2076-x

[32] Roskoski R. The ErbB/HER family of protein-tyrosine kinases and cancer. Pharmacol Res 2014;79:34–74. 24269963 10.1016/j.phrs.2013.11.002

[33] Yan L, Yang M, Guo H et al. Single-cell RNA-Seq profiling of human preimplantation embryos and embryonic stem cells. Nat Struct Mol Biol 2013;20:1131–9. 23934149 10.1038/nsmb.2660

[34] Xiong Z, Xu K, Lin Z et al. Ultrasensitive Ribo-seq reveals translational landscapes during mammalian oocyte-to-embryo transition and pre-implantation development. Nat Cell Biol 2022;24:968–80. 35697785 10.1038/s41556-022-00928-6

[35] Yu X, Liang S, Chen M et al. Recapitulating early human development with 8C-like cells. Cell Rep 2022;39:110994. 35732112 10.1016/j.celrep.2022.110994

[36] Xue Z, Huang K, Cai C et al. Genetic programs in human and mouse early embryos revealed by single-cell RNA sequencing. Nature 2013;500:593–7. 23892778 10.1038/nature12364 PMC4950944

[37] Collier AJ, Bendall A, Fabian C et al. Genome-wide screening identifies Polycomb repressive complex 13 as an essential regulator of human naïve pluripotent cell reprogramming. Sci Adv 2022;8:eabk0013. 35333572 10.1126/sciadv.abk0013 PMC8956265

[38] Doench JG. Am I ready for CRISPR? A user’s guide to genetic screens. Nat Rev Genet 2018;19:67–80. 29199283 10.1038/nrg.2017.97

[39] Bayerl J, Ayyash M, Shani T et al. Principles of signaling pathway modulation for enhancing human naive pluripotency induction. Cell Stem Cell 2021;28:1549–1565.e12. 33915080 10.1016/j.stem.2021.04.001 PMC8423434

[40] Tebbutt N, Pedersen MW, Johns TG. Targeting the ERBB family in cancer: couples therapy. Nat Rev Cancer 2013;13:663–73. 23949426 10.1038/nrc3559

[41] Che M, Chaturvedi A, Munro SA et al. Opposing transcriptional programs of KLF5 and AR emerge during therapy for advanced prostate cancer. Nat Commun 2021;12:6377. 34737261 10.1038/s41467-021-26612-1 PMC8568894

[42] Park JM, Wu T, Cyr AR et al. The role of Tcfap2c in tumorigenesis and cancer growth in an activated Neu model of mammary carcinogenesis. Oncogene 2015;34:6105–14. 25772240 10.1038/onc.2015.59 PMC4573379

[43] Kinisu M, Choi YJ, Cattoglio C et al. Klf5 establishes bi-potential cell fate by dual regulation of ICM and TE specification genes. Cell Rep 2021;37:109982. 34758315 10.1016/j.celrep.2021.109982 PMC8711565

[44] Zhu M, Cornwall-Scoones J, Wang P et al. Developmental clock and mechanism of *de novo* polarization of the mouse embryo. Science 2020;370:eabd2703. 33303584 10.1126/science.abd2703 PMC8210885

