# Supplementary material for: A CRISPR/Cas9-based kinome screen identifies ErbB signaling as a new regulator of human naïve pluripotency and totipotency
Source: Life Med. 2023 Oct 20;2(4):lnad037. doi: 10.1093/lifemedi/lnad037 (PMC11749542; doi:10.1093/lifemedi/lnad037)
Supplement: lnad037_suppl_Supplementary_Figures [file lnad037_suppl_Supplementary_Figures.pdf]

Figure S1

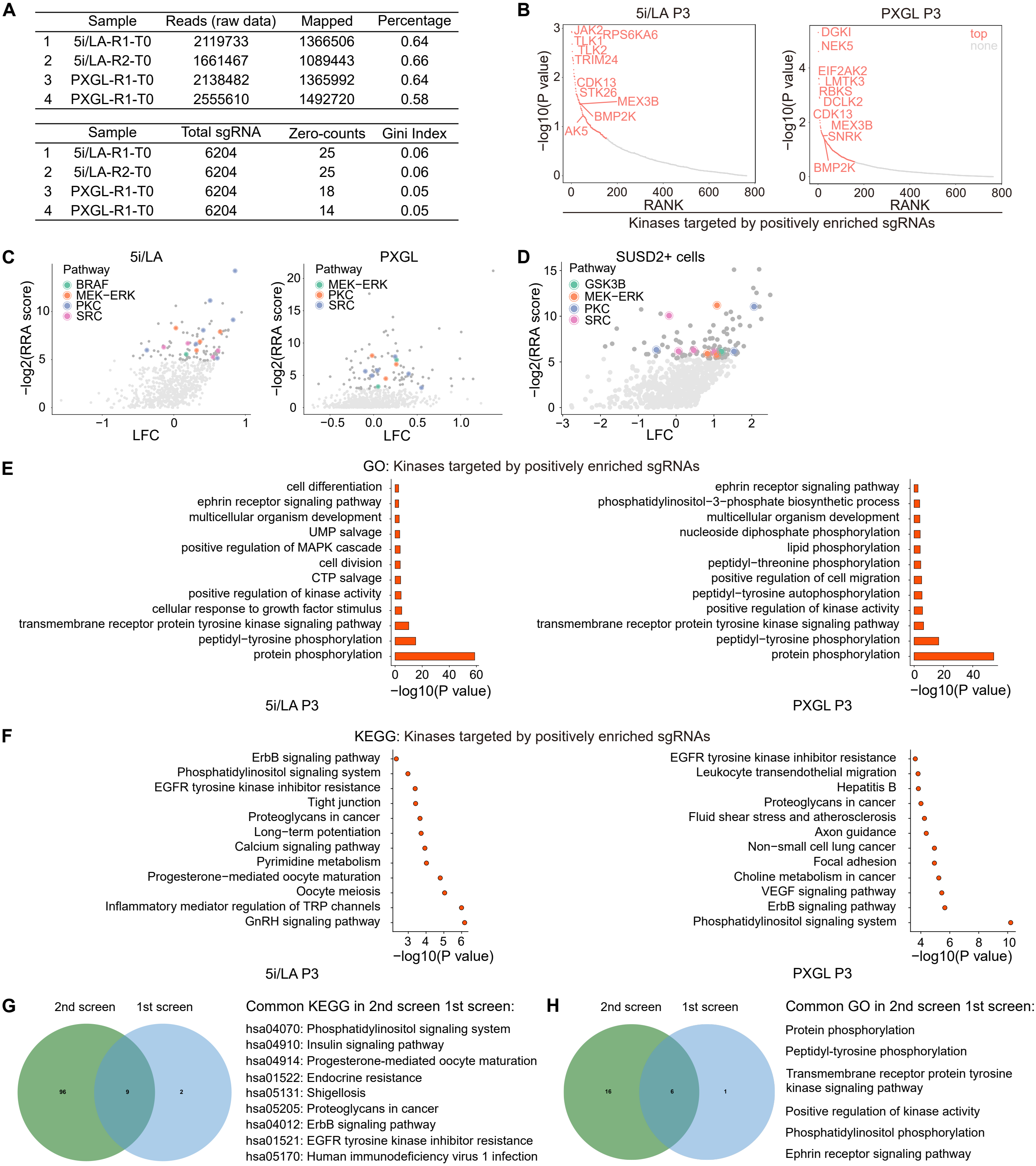

	Sample	Reads (raw data)	Mapped	Percentage
1	5i/LA-R1-T0	2119733	1366506	0.64
2	5i/LA-R2-T0	1661467	1089443	0.66
3	PXGL-R1-T0	2138482	1365992	0.64
4	PXGL-R1-T0	2555610	1492720	0.58

	Sample	Total sgRNA	Zero-counts	Gini Index
1	5i/LA-R1-T0	6204	25	0.06
2	5i/LA-R2-T0	6204	25	0.06
3	PXGL-R1-T0	6204	18	0.05
4	PXGL-R1-T0	6204	14	0.05

Figure S1. Further analysis on CRISPR kinome KO screening result

A. The statistics of mapped percentage of sgRNA reads and Gini index of sgRNA Zero-counts after transited from XF-LCDM condition to PXGL and 5i/LA conditions.
B. Kinases targeted by positively enriched sgRNAs at passage 3 under naïve pluripotent condition 5i/LA (left) and PXGL (right).
C. Scatter plot analysis of the enrichment of known naïve pluripotent related kinases at passage 6 under 5i/LA (left) and PXGL (right).
D. Scatter plot analysis of the enrichment of known naïve pluripotent related kinases in sorted SUSD2 positve cells at passage 3 under XF-LCDM condition.
E. GO analysis of kinases targeted by positively enriched sgRNAs in cells under 5i/LA (passage 3) (left), PXGL (passage 3) (right).
F. KEGG analysis of kinases targeted by positively enriched sgRNAs in cells under 5i/LA (passage 3) (left), PXGL (passage 3) (right).
G. Venn diagram of enriched KEGG signalling pathways for the screens with two strategies. Commonly enriched terms are listed.
H. Venn diagram of enriched GO BP terms for the screens with two strategies. Commonly enriched pathways are listed.

Figure S2

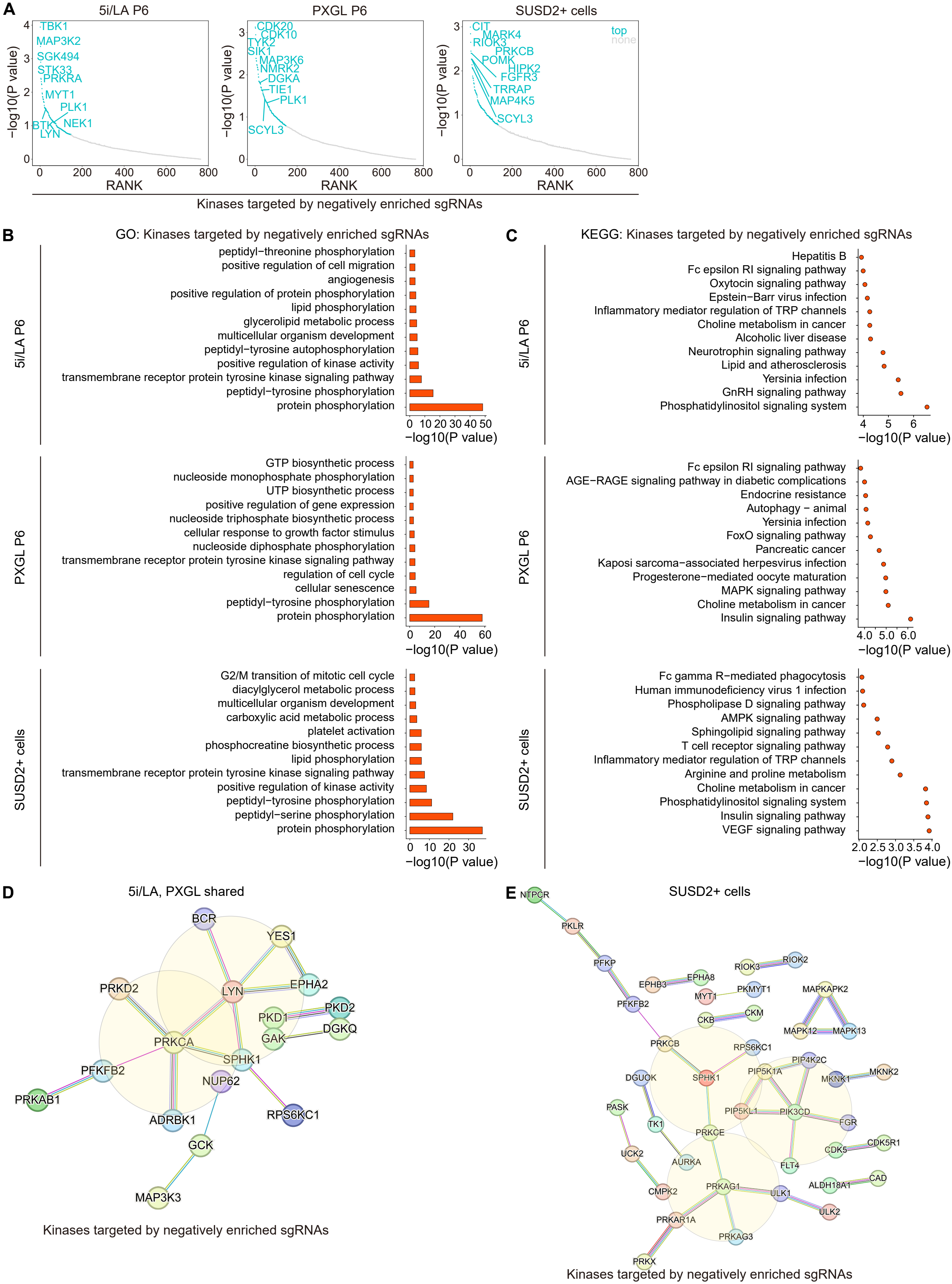
A
5i/LA P6
PXGL P6
SUSD2+ cells
−log10(P value)
RANK
top
none
TBK1
MAP3K2
SGK494
STK33
PRKRA
MYT1
PLK1
NEK1
BTK
LYN
CDK20
CDK10
TYK2
SIK1
MAP3K6
NMRK2
DGKA
TIE1
PLK1
SCYL3
CIT
MARK4
RIOK3
PRKCB
POMK
HIPK2
FGFR3
TRRAP
MAP4K5
SCYL3
Kinases targeted by negatively enriched sgRNAs
B
GO: Kinases targeted by negatively enriched sgRNAs
5i/LA P6
peptidyl−threonine phosphorylation
positive regulation of cell migration
angiogenesis
positive regulation of protein phosphorylation
lipid phosphorylation
glycerolipid metabolic process
multicellular organism development
peptidyl−tyrosine autophosphorylation
positive regulation of kinase activity
transmembrane receptor protein tyrosine kinase signaling pathway
peptidyl−tyrosine phosphorylation
protein phosphorylation
PXGL P6
GTP biosynthetic process
nucleoside monophosphate phosphorylation
UTP biosynthetic process
positive regulation of gene expression
nucleoside triphosphate biosynthetic process
cellular response to growth factor stimulus
nucleoside diphosphate phosphorylation
transmembrane receptor protein tyrosine kinase signaling pathway
regulation of cell cycle
cellular senescence
peptidyl−tyrosine phosphorylation
protein phosphorylation
SUSD2+ cells
G2/M transition of mitotic cell cycle
diacylglycerol metabolic process
multicellular organism development
carboxylic acid metabolic process
platelet activation
phosphocreatine biosynthetic process
lipid phosphorylation
transmembrane receptor protein tyrosine kinase signaling pathway
positive regulation of kinase activity
peptidyl−tyrosine phosphorylation
peptidyl−serine phosphorylation
protein phosphorylation
−log10(P value)
C
KEGG: Kinases targeted by negatively enriched sgRNAs
5i/LA P6
Hepatitis B
Fc epsilon RI signaling pathway
Oxytocin signaling pathway
Epstein−Barr virus infection
Inflammatory mediator regulation of TRP channels
Choline metabolism in cancer
Alcoholic liver disease
Neurotrophin signaling pathway
Lipid and atherosclerosis
Yersinia infection
GnRH signaling pathway
Phosphatidylinositol signaling system
PXGL P6
Fc epsilon RI signaling pathway
AGE−RAGE signaling pathway in diabetic complications
Endocrine resistance
Autophagy − animal
Yersinia infection
FoxO signaling pathway
Pancreatic cancer
Kaposi sarcoma−associated herpesvirus infection
Progesterone−mediated oocyte maturation
MAPK signaling pathway
Choline metabolism in cancer
Insulin signaling pathway
SUSD2+ cells
Fc gamma R−mediated phagocytosis
Human immunodeficiency virus 1 infection
Phospholipase D signaling pathway
AMPK signaling pathway
Sphingolipid signaling pathway
T cell receptor signaling pathway
Inflammatory mediator regulation of TRP channels
Arginine and proline metabolism
Choline metabolism in cancer
Phosphatidylinositol signaling system
Insulin signaling pathway
VEGF signaling pathway
−log10(P value)
D
5i/LA, PXGL shared
BCR
YES1
PRKD2
LYN
EPHA2
PKD1
PKD2
DGKQ
GAK
PRKCA
SPHK1
PFKFB2
NUP62
PRKAB1
ADRBK1
RPS6KC1
GCK
MAP3K3
Kinases targeted by negatively enriched sgRNAs
E
SUSD2+ cells
NTPCR
PKLR
PFKP
RIOK3
RIOK2
EPHB3
EPHA8
MYT1
PKMYT1
MAPKAPK2
PFKFB2
CKB
CKM
MAPK12
MAPK13
PRKCB
RPS6KC1
PIP4K2C
DGUOK
SPHK1
PIP5K1A
MKNK1
MKNK2
PASK
PIP5KL1
PIK3CD
FGR
TK1
PRKCE
CDK5
CDK5R1
UCK2
AURKA
FLT4
ALDH18A1
CAD
CMPK2
PRKAG1
ULK1
ULK2
PRKAR1A
PRKAG3
PRKX
Kinases targeted by negatively enriched sgRNAs

Figure S2. Analysis of underrepresented kinases during the transition towards naïve pluripotency

A. Kinases targeted by negatively enriched sgRNAs in cells at passage 6 under naïve pluripotent conditions 5i/LA (left), PXGL (middle) and sorted SUSD2 positive cells at passage 3 under the XF-LCDM condition (right). Top enriched genes and commonly enriched genes under two conditions are shown.

B. GO analysis of kinases targeted by negatively enriched sgRNAs in cells under 5i/LA (passage 6) (upper), PXGL (passage 6) (middle), and sorted SUSD2+ cells at passage 3 under the XF-LCDM condition (lower).

C. KEGG analysis of kinases targeted by negatively enriched sgRNAs in cells under 5i/LA (passage 6) (upper), PXGL (passage 6) (middle), and sorted SUSD2+ cells at passage 3 under the XF-LCDM condition (lower).

D. Protein-protein interaction analysis of kinases targeted by negatively enriched sgRNAs in cells under PXGL and 5i/LA conditions from the first screen. Color of lines between nodes indicated evidence types. Interaction score = 0.700.

E. Protein-protein interaction analysis of kinases targeted by negatively enriched sgRNAs in sorted SUSD2 positive cells under the XF-LCDM condition. Color of lines between nodes indicated evidence types. Interaction score = 0.700.

Figure S3

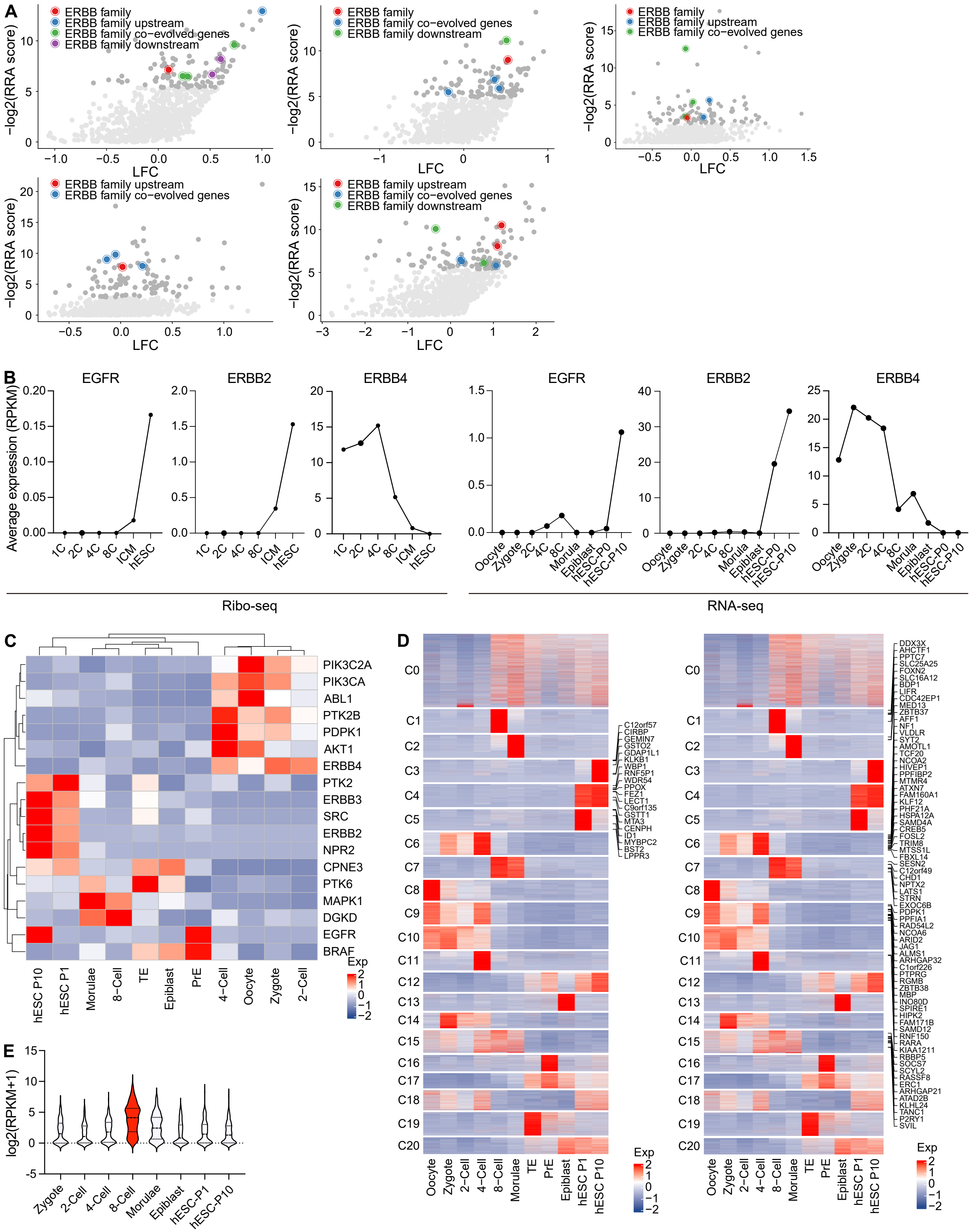
A
ERBB family
ERBB family upstream
ERBB family co−evolved genes
ERBB family downstream
ERBB family co−evolved genes
−log2(RRA score)
LFC
B
EGFR
ERBB2
ERBB4
Average expression (RPKM)
1C
2C
4C
8C
ICM
hESC
Oocyte
Zygote
Morula
Epiblast
hESC-P0
hESC-P10
Ribo-seq
RNA-seq
C
PIK3C2A
PIK3CA
ABL1
PTK2B
PDPK1
AKT1
ERBB4
PTK2
ERBB3
SRC
ERBB2
NPR2
CPNE3
PTK6
MAPK1
DGKD
EGFR
BRAF
hESC P10
hESC P1
Morulae
8−Cell
TE
Epiblast
PrE
4−Cell
Oocyte
Zygote
2−Cell
Exp
D
C0
C1
C2
C3
C4
C5
C6
C7
C8
C9
C10
C11
C12
C13
C14
C15
C16
C17
C18
C19
C20
C12orf57
CIRBP
GEMIN7
GSTO2
GDAP1L1
KLKB1
WBP1
RNF5P1
WDR54
PPOX
FEZ1
LECT1
C9orf135
GSTT1
MTA3
CENPH
ID1
MYBPC2
BST2
LPPR3
DDX3X
AHCTF1
PPTC7
SLC25A25
FOXN2
SLC16A12
BDP1
LIFR
CDC42EP1
MED13
ZBTB37
AFF1
NF1
VLDLR
SYT2
AMOTL1
TCF20
NCOA2
HIVEP1
PPFIBP2
MTMR4
ATXN7
FAM160A1
KLF12
PHF21A
HSPA12A
SAMD4A
CREB5
FOSL2
TRIM8
MTSS1L
FBXL14
SESN2
C12orf49
CHD1
NPTX2
LATS1
STRN
EXOC6B
PDPK1
PPFIA1
RAD54L2
NCOA6
ARID2
JAG1
ALMS1
ARHGAP32
C1orf226
PTPRG
RGMB
ZBTB38
MBP
INO80D
SPIRE1
HIPK2
FAM171B
SAMD12
RNF150
RARA
KIAA1211
RBBP5
SOCS7
SCYL2
RASSF8
ERC1
ARHGAP21
ATAD2B
KLHL24
TANC1
P2RY1
SVIL
Oocyte
Zygote
2−Cell
4−Cell
8−Cell
Morulae
TE
PrE
Epiblast
hESC P1
hESC P10
Exp
E
log2(RPKM+1)
Zygote
2-Cell
4-Cell
8-Cell
Morulae
Epiblast
hESC-P1
hESC-P10

Figure S3. Further analysis on ErbB signalling promoting human totipotency

A. Scatter plot analysis of the enrichment of ErbB family genes and their related genes in our CRISPR kinome KO screen.
B. Line chart showing the expression of EGFR, ERBB2 and ERBB4 at different developmental stages based on published RNA-sequencing data (Liying Yan et al., 2013) and RIBO- sequencing data (Zhuqing Xiong et al., 2022).
C. Heatmap analysis showing the expression of all kinases belonging to the ERBB signalling pathway at different developmental stages based on published RNA-sequencing data (Liying Yan et al., 2013).
D. Heatmap of clustering result of published RNA-sequencing data (Liying Yan et al., 2013). Genes significantly up-regulated in primed pluripotent stem cells are listed in the left panel, which are also marked in the volcano plots in Figure 4. Pan-early embryonic genes from cluster C1, C2, C6, C7, C9, C11, and C15 are listed in the right panel, which are also marked in the volcano plots in Figure 4.
E. Violin plot showing the expression levels of the 8C specific genes at different developmental stages based on published RNA-sequencing data (Liying Yan et al., 2013), which is related to Figure 4. The values are represented as $\log_2$ (RPKM + 1).

Figure S4

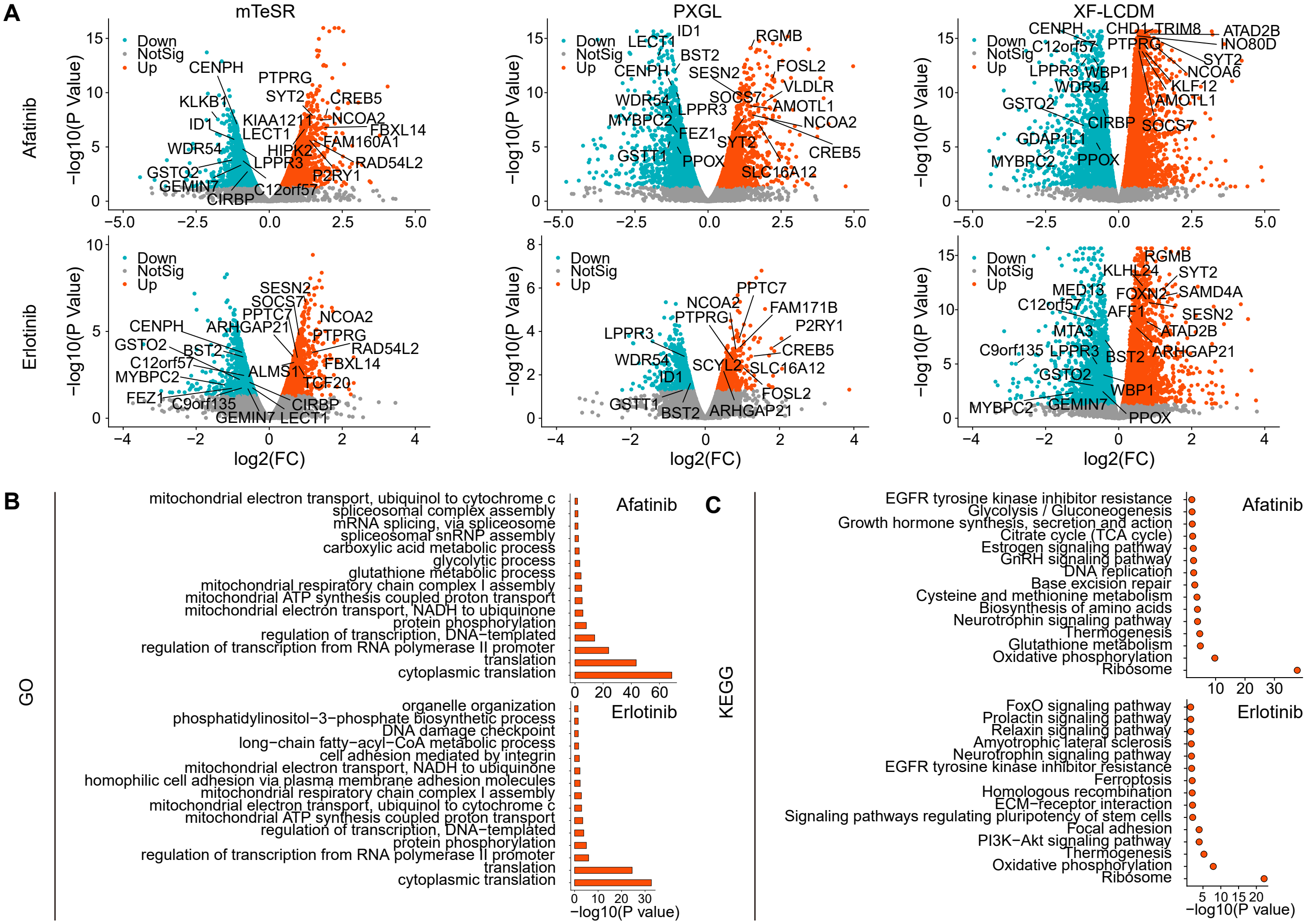

A
mTeSR
PXGL
XF-LCDM
Afatinib
Erlotinib
Down
NotSig
Up
−log10(P Value)
log2(FC)
B
GO
Afatinib
mitochondrial electron transport, ubiquinol to cytochrome c
spliceosomal complex assembly
mRNA splicing, via spliceosome
spliceosomal snRNP assembly
carboxylic acid metabolic process
glycolytic process
glutathione metabolic process
mitochondrial respiratory chain complex I assembly
mitochondrial ATP synthesis coupled proton transport
mitochondrial electron transport, NADH to ubiquinone
protein phosphorylation
regulation of transcription, DNA−templated
regulation of transcription from RNA polymerase II promoter
translation
cytoplasmic translation
Erlotinib
organelle organization
phosphatidylinositol−3−phosphate biosynthetic process
DNA damage checkpoint
long−chain fatty−acyl−CoA metabolic process
cell adhesion mediated by integrin
mitochondrial electron transport, NADH to ubiquinone
homophilic cell adhesion via plasma membrane adhesion molecules
mitochondrial respiratory chain complex I assembly
mitochondrial electron transport, ubiquinol to cytochrome c
mitochondrial ATP synthesis coupled proton transport
regulation of transcription, DNA−templated
protein phosphorylation
regulation of transcription from RNA polymerase II promoter
translation
cytoplasmic translation
−log10(P value)
C
KEGG
Afatinib
EGFR tyrosine kinase inhibitor resistance
Glycolysis / Gluconeogenesis
Growth hormone synthesis, secretion and action
Citrate cycle (TCA cycle)
Estrogen signaling pathway
GnRH signaling pathway
DNA replication
Base excision repair
Cysteine and methionine metabolism
Biosynthesis of amino acids
Neurotrophin signaling pathway
Thermogenesis
Glutathione metabolism
Oxidative phosphorylation
Ribosome
Erlotinib
FoxO signaling pathway
Prolactin signaling pathway
Relaxin signaling pathway
Amyotrophic lateral sclerosis
Neurotrophin signaling pathway
EGFR tyrosine kinase inhibitor resistance
Ferroptosis
Homologous recombination
ECM−receptor interaction
Signaling pathways regulating pluripotency of stem cells
Focal adhesion
PI3K−Akt signaling pathway
Thermogenesis
Oxidative phosphorylation
Ribosome
−log10(P value)

Figure S4. Further transcriptomic analysis of ErbB family inhibitors induced naïve pluripotency and totipotent features

A. Volcano plot showing differentially expressed genes between Afatinib (upper panels), Erlotinib (lower panels) treatment and non-treated control under mTeSR (left), PXGL (middle) and PXGL (right) conditions. Significantly enriched genes specific to primed pluripotency marker genes and pan-early embryonic genes are marked.
B. GO terms enriched upon Afatinib (upper) and Erlotinib (lower) treatment under PXGL condition.
C. KEGG signalling pathways enriched upon Afatinib (upper) and Erlotinib (lower) treatment under PXGL condition.
