# Supplementary material for: A CRISPR/Cas9-based kinome screen identifies ErbB signaling as a new regulator of human naïve pluripotency and totipotency
Source: Life Med. 2023 Oct 20;2(4):lnad037. doi: 10.1093/lifemedi/lnad037 (PMC11749542; doi:10.1093/lifemedi/lnad037)
Supplement: lnad037_suppl_Supplementary_Data [file lnad037_suppl_Supplementary_Data.docx]

**Table S1**

sgRNA list of all human kinases and sequences of non-target controls used in our screen.

**Table S2**

Statistical results for CRISPR kinome KO screen.

**Table S3**

Chemical molecule list for screening validation.

**Table S4**

Normalized FPKM and GSEA analysis for Bulk RNA sequencing result.

**Table S5**

8C specific genes used in this study.

**Table S6**

RT-qPCR primers and Primer sequences for amplifying sgRNA library and sequencing.
